# Human forebrain organoids-based multi-omics analyses reveal PCCB’s regulation on GABAergic system contributing to schizophrenia

**DOI:** 10.21203/rs.3.rs-2674668/v1

**Published:** 2023-03-29

**Authors:** Wendiao Zhang, Ming Zhang, Zhenhong Xu, Hongye Yan, Huimin Wang, Jiamei Jiang, Juan Wan, Beisha Tang, Chunyu Liu, Chao Chen, Qingtuan Meng

**Affiliations:** Center for Medical Genetics & Hunan Key Laboratory of Medical Genetics, School of Life Sciences, Central South University; School of Life Sciences, Central South University; The First Affiliated Hospital of University of South China; The First Affiliated Hospital of University of South China; The First Affiliated Hospital of University of South China; The First Affiliated Hospital of University of South China; The First Affiliated Hospital of University of South China; Xiangya Hospital, Central South University; SUNY Upstate Medical University; Central South University; The First Affiliated Hospital of University of South China

## Abstract

Identifying genes whose expression is associated with schizophrenia (SCZ) risk by transcriptome-wide association studies (TWAS) facilitates downstream experimental studies. Here, we integrated multiple published datasets of TWAS (including FUSION, PrediXcan, summary-data-based Mendelian randomization (SMR), joint-tissue imputation approach with Mendelian randomization (MR-JTI)), gene coexpression, and differential gene expression analysis to prioritize SCZ candidate genes for functional study. Convergent evidence prioritized Propionyl-CoA Carboxylase Subunit Beta *(PCCB)*, a nuclear-encoded mitochondrial gene, as an SCZ risk gene. However, the *PCCB’s* contribution to SCZ risk has not been investigated before. Using dual luciferase reporter assay, we identified that SCZ-associated SNP rs35874192, an eQTL SNP for *PCCB*, showed differential allelic effects on transcriptional activities. *PCCB* knockdown in human forebrain organoids (hFOs) followed by RNA-seq revealed dysregulation of genes enriched with multiple neuronal functions including gamma-aminobutyric acid (GABA)-ergic synapse, as well as genes dysregulated in postmortem brains of SCZ patients or in cerebral organoids derived from SCZ patients. The metabolomic and mitochondrial function analyses confirmed the deceased GABA levels resulted from reduced tricarboxylic acid cycle in *PCCB* knockdown hFOs. Multielectrode array recording analysis showed that *PCCB* knockdown in hFOs resulted into SCZ-related phenotypes including hyper-neuroactivities and decreased synchronization of neural network. In summary, this study utilized hFOs-based multi-omics data and revealed that *PCCB* downregulation may contribute to SCZ risk through regulating GABAergic system, highlighting the mitochondrial function in SCZ.

## Introduction

Schizophrenia (SCZ) is a complex polygenic psychiatric disorder with risk contributed by environmental and genetic factors^1^. Genetic studies such as genome-wide association studies (GWAS) have identified hundreds of common single nucleotide polymorphisms (SNPs) associated with SCZ^2,3^. Most of the SCZ-associated SNPs are non-coding variants located in regulatory DNA elements^4–6^, suggesting that gene expression mediates the connection between genetic variants and SCZ phenotypes^7^. Identifying genes whose expression is associated with SCZ phenotypes facilitates discovering SCZ risk genes for downstream functional studies.

By integrating SCZ GWAS and brain expression quantitative trait loci (eQTL) data, several approaches which are collectively described as transcriptome-wide association studies (TWAS) have been used to identify SCZ risk genes. These TWAS approaches, including FUSION^8–10^, PrediXcan^11^, summary-data-based Mendelian randomization (SMR)^2,10,12,13^, and joint-tissue imputation approach with Mendelian randomization (MR-JTI)^14^, aimed to identify the association between predicted gene expression and SCZ risk. Though MR-JTI could improve gene expression prediction performance in TWAS and provide a causal inference framework^15^, experimental validation is still needed.

Here we integrated results from MR-JTI^14^ and other published SCZ TWAS datasets^2,8–13^ to prioritize SCZ risk genes for functional study. Through these procedures, we identified Propionyl-CoA Carboxylase Subunit Beta *(PCCB)*, a protein-coding gene that plays important roles in mitochondrial metabolism^16,17^, as an SCZ risk gene with the most supporting evidence in our analysis. However, the *PCCB’s* contribution to SCZ risk has not been investigated before. Using human forebrain organoids (hFOs), three-dimensional cell cultures that capture key aspects of human brain^18^, we found that *PCCB* knockdown in hFOs resulted into SCZ pathology-related cellular phenotypes. We also identified that SCZ-associated SNP rs35874192 may regulate *PCCB* expression, supporting that SCZ-associated common genetic variants may regulate *PCCB* expression which mediates the genetic effects on SCZ risk.

## Results

### PCCB is prioritized as a promising SCZ risk gene

To obtain reliable SCZ risk genes for downstream functional study, we integrated multiple published TWAS datasets (**Table S1**) to prioritize genes with sufficient supporting evidence. We also checked whether the prioritized genes are located in SCZ risk-associated gene coexpression module or dysregulated in postmortem SCZ brains. These analyses prioritized *PCCB, GATAD2A*, and *GNL3* as the top three SCZ risk genes (Table 1). Notably, *PCCB* was also identified as an SCZ risk gene in the gene-based MAGMA analysis^19^. Moreover, *PCCB* is located in the gene coexpression module (M2) that is downregulated in SCZ based on the PsychENCODE data^10^. *PCCB* was also found to be nominally downregulated in postmortem SCZ brains (P = 0.01, FDR = 0.14) in the CommonMind data^20^. These lines of evidence suggested that *PCCB* expression mediated the genetic effects on SCZ risk. Therefore, we focused on studying how *PCCB* contributes to SCZ risk in this study.

### PCCB eQTL SNP rs35874192 affects transcriptional activities

Since *PCCB* expression is genetically associated with SCZ, we investigated the functional impacts of SCZ-associated SNPs on *PCCB* expression. Based on the TWAS results used in this study, we retrieved the top SNPs (rs7432375, rs7427564, rs527888, rs66691851) and their linkage disequilibrium (LD) SNPs that were associated with *PCCB* expression. To narrow down to the putatively causal variants, we focused on those eQTL SNPs (eSNPs) that are likely to affect *PCCB* expression in the brain. Since opening chromatin facilitates gene expression activation, we used brain ATAC-seq data from the PsychENCODE consortium^21^ to identify eSNPs located in active transcription regions. By integrating PsychENCODE ATAC-seq data and SNP annotation information from the Roadmap Epigenetics Consortium^22^, we prioritized three eSNPs (rs35874192, rs900818, rs7349597) (Table 2) that are located in genomic regions strongly suggested as enhancers or promoters in the human brain tissues or neural cell cultures.

**Figure 1:**
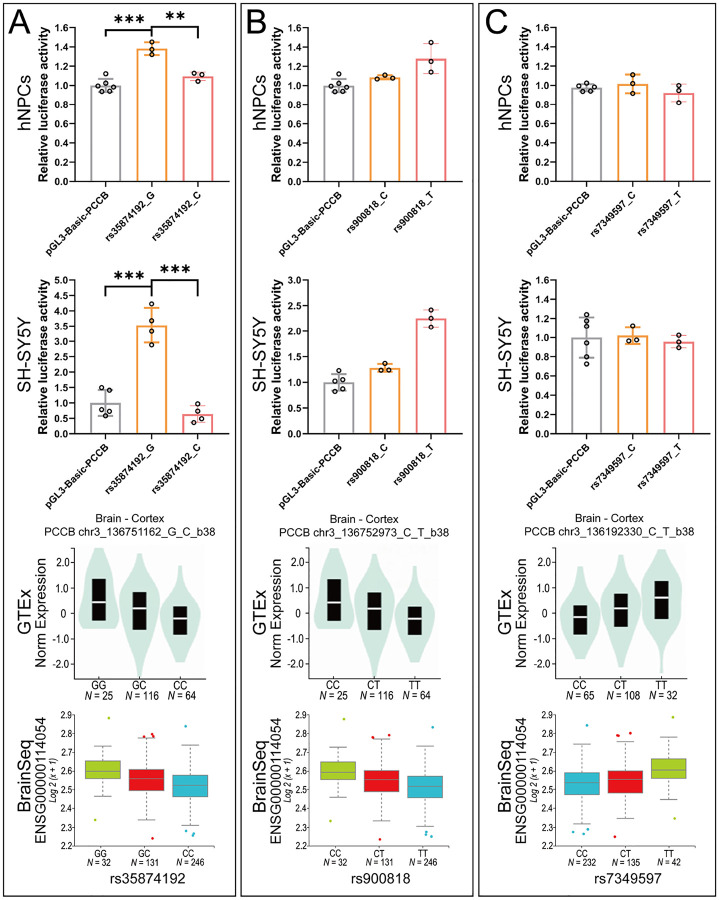
DLRA in hNPCs and SH-SY5Y cell lines. A, B, and C DLRA results for *PCCB* eSNPs rs35874192 (A), rs900818 (B), and rs7349597 (C) in hNPCs and SH-SY5Y. The PRL-TK Renilla vector was used as internal control. Data are shown as Mean ± SEM. Unpaired two tailed t test was used for comparison between two groups. **P< 0.01, ***P< 0.001. Reference eQTL plots in this figure were downloaded from the GTEx portal (https://www.gtexportal.org/home/) and BrainSeq phaseleQTL data (http://eqtl.brainseq.org/phase1/eqtl/).

We then performed dual luciferase reporter assay (DLRA) in both hNPCs and SH-SY5Y cell lines to validate the regulatory effects of the three eSNP-containing DNA elements. For each eSNP 50 base pairs (bp) eSNP-containing DNA fragment was synthesized and cloned into the upstream of *PCCB* promoter in the PGL3-basic luciferase reporter vector (Fig. S1). In DLRA, the eSNP rs35874192 (G/C) showed allelic effects on transcriptional activities in both hNPCs and SH-SY5Y cell lines, with SCZ-associated allele C corresponding to a lower gene expression (Fig. 1A). Notably, the directions of allelic effects of rs35874192 were consistent with eQTL patterns detected in brain tissues from the GTEx^23^ and BrainSeq consortium^24^ (Fig. 1A). While the eSNPs rs900818 and rs7349597 had no differential allelic effects on transcriptional activities (Fig. 1B, C).

### PCCB knockdown in hFOs affects expression of genes enriched in GABAergic synapse

**Figure 2:**
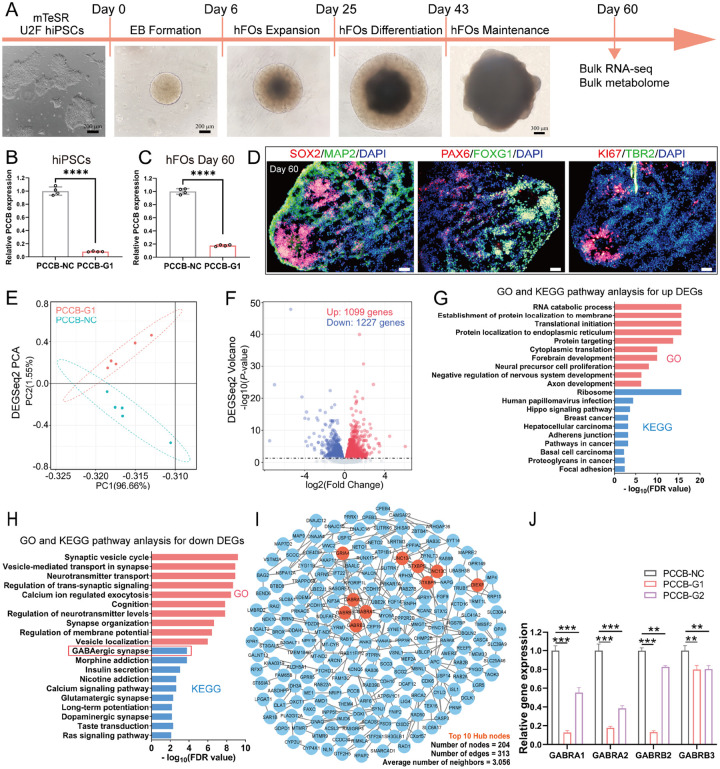
Functional effects of *PCCB* knockdown in hFOs. A Workflow of hFOs culture in this study. Scale bar, 200 μm and 300 μm. B, C RT-qPCR analysis for *PCCB* expression in hiPSCs (B) and hFOs (C). D Immunostaining characterization for hFOs. Cortical plate marker, MAP2; intermediate zone marker, TBR2; ventricular zone markers, SOX2 and ki67; forebrain-speciflc markers, FOXG1 and PAX6. Scale bar, 50 μm. EPCA plot for the *PCCB* knockdown and control hFOs. *PCCB*-NC was shown with green dots and PCCB-G1 was shown with red dots. F Volcano plot of DEGs between the *PCCB* knockdown and control hFOs. Upregulated genes are shown with red dots and downregulated genes are shown with blue dots. G, H GO and KEGG analysis for *PCCB*-induced upregulated (G) and downregulated DEGs (H) respectively. GO terms are shown with red bars, and KEGG are shown with blue bars. I PPI network analysis for 350 shared *PCCB*-induced downregulated genes between PCCB-G1 and *PCCB-G2* hFOs. The top 10 hub nodes are shown with orange nodes. J RT-qPCR analysis for *GABRA1, GABRA2, GABRB2*, and *GABRB3* (The hub nodes in the PPI network). The two-tailed student’s t-test was used to assess difference between the *PCCB-NC* and PCCB-G1 or *PCCB-G2* group. **P< 0.01, ***P< 0.001, ****P< 0.0001.

According to the TWAS and DLRA results, lower *PCCB* expression is associated with increased SCZ risk. We established *PCCB* knockdown and control human induced pluripotent stem cells (hiPSCs, U2F) using CRISPR interference (CRISPRi). In CRISPRi, one guide RNA (gRNA) sequence targeting *PCCB* (PCCB-G1) and one non-targeting control gRNA were designed. The established *PCCB* knockdown and control hiPSCs were then used to generate hFOs (Fig. 2A, B) to investigate the functional impacts of *PCCB* knockdown. On day 60 of organoid culture (Fig. 2A, C, D), *PCCB* knockdown and control hFOs were used for RNA-sequencing (RNA-seq). Differential gene expression analysis identified 2326 differentially expressed genes (DEGs) [false discovery rate (FDR) < 0.05] between the *PCCB* knockdown and control hFOs (Table S2). Among the 2326 DEGs, 1099 genes were upregulated and 1227 genes were downregulated in *PCCB* knockdown hFOs (Fig. 2E, F).

We next used WebGestalt 2019^25^ to annotate biological functions of the *PCCB*-induced DEGs. For the upregulated DEGs, Gene Ontology (GO) analysis revealed their significant enrichment in biological functions including RNA catabolic process (FDR< 2.20E-16), forebrain development (FDR = 1.11 E-10), and neural precursor cell proliferation (FDR = 8.96E-09). The Kyoto Encyclopedia of Genes and Genomes (KEGG) pathway analysis implicated the upregulated DEGs in the ribosome (FDR< 2.20E-16) and hippo signaling (FDR = 2.43E-04) pathways (Fig. 2G and **Table S2**). For the downregulated DEGs, GO analysis showed that they were enriched with neuronal functions, including synaptic vesicle cycle (FDR = 6.28E-10), neurotransmitter transport (FDR = 1.23E-09), and synapse organization (FDR = 2.27E-07). The KEGG pathway analysis implicated the downregulated DEGs in the gamma-aminobutyric acid (GABA)-ergic synapse (FDR = 1.73E-04), morphine addiction (FDR = 1.73E-04), and nicotine addiction (FDR = 2.31 E-03) pathways (Fig. 2H and **Table S2**). These results highlighted that reduced expression of *PCCB* may downregulate genes related to neuronal functions and GABAergic synapse.

To confirm the RNA-seq results and to minimize potential off-target effects in CRISPRi, we generated another *PCCB* knockdown hFOs using a second gRNA (PCCB-G2) and performed RNA-seq (**Fig. S2**). We found that 1079 of 2326 DEGs in *PCCB*-G1 hFOs overlapped with those identified in *PCCB*-G2 hFOs. The 1079 overlapped DEGs were also enriched with neuronal functions such as synapse organization (FDR = 5.66E-04), forebrain development (FDR = 1.13E-02), and axon development (FDR = 1.51E-02) (**Fig. S2** and **Table S2**). Of the 1079 overlapped DEGs, 350 genes were downregulated in both *PCCB*-G1 and *PCCB*-G2 hFOs. Using the shared 350 downregulated DEGs, we constructed a protein-protein interaction (PPI) network and found that GABA receptor genes including *GABRA1, GABRA2, GABRB2*, and *GABRB3* were hub nodes in the PPI network (Fig. 2I). Real-time quantitative PCR (RT-qPCR) further confirmed the decreased expression of these GABA receptor genes in *PCCB* knockdown hFOs (Fig. 2J). These results further highlighted that *PCCB* may affect the GABAergic system.

### PCCB -induced DEGs in hFOs are enriched with SCZ-related genes

To explore *PCCB*’s connection to SCZ, we evaluated the enrichment of 1079 PCCB-induced DEGs in hFOs with SCZ-related gene sets. The first SCZ gene set was 4096 differentially expressed protein-coding genes between postmortem brains of 559 SCZ patients and 936 controls from the PsychENCODE consortium^10^. The second SCZ gene set was 2809 DEGs between cerebral organoids (6 months) derived from eight SCZ patients and eight controls from the Kathuria et al. study^26^. We found that the 1079 *PCCB*-induced DEGs were significantly overlapped with genes dysregulated in PsychENCODE SCZ brains (Overlapped genes = 282, *P* = 5.84E-04) and SCZ patient-derived cerebral organoids (Overlapped genes = 255, *P* = 1.92E-14) (**Fig. S3A**). We also found that the 1079 *PCCB*-induced DEGs were significantly *(P*_*adjust*_ = 7.09E-3) overlapped with genes reported in SCZ GWAS from the FUMA analysis^27^ (**Fig. S3B**). These results suggested that *PCCB* may contribute to SCZ risk by affecting expression of genes related to SCZ (**Table S2**).

### Metabolic analysis confirms the decreased GABA level in PCCB knockdown hFOs

**Figure 3:**
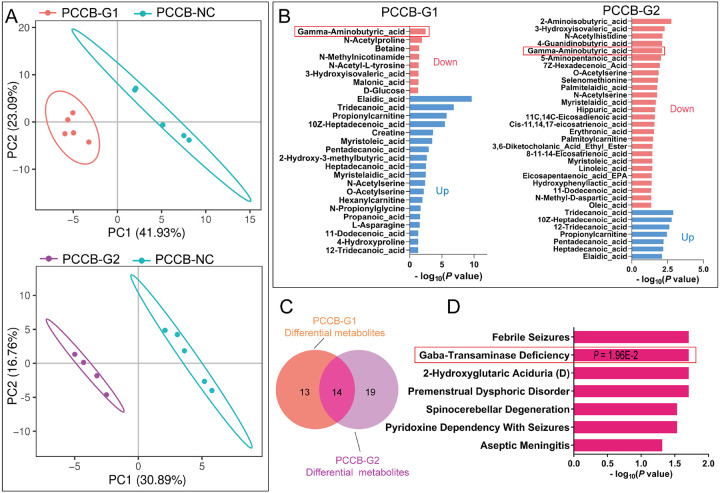
Metabolomic analysis after *PCCB* knockdown in hFOs. A PCA plot for *PCCB* metabolomic analysis in hFOs. BMetabolomic analysis showed that there were 27 and SS differentially expressed metabolites in *PCCB*-G1 and *PCCB-G2*, respectively. Among them, GABA was downregulated significantly in both groups (red box). C Overlap for differential metabolites identified in *PCCB*-G1 and *PCCB-G2* hFOs. D functional annotation for 14 shared differential metabolites.

RNA-seq analysis revealed the effects of *PCCB* knockdown on GABAergic synapse, we further used metabolomic analysis to examine whether *PCCB* knockdown decreased GABA levels in hFOs. Of the 178 detected metabolites in hFOs (day 60), 27 and 33 metabolites were differentially expressed in *PCCB*-G1 and *PCCB*-G2 hFOs (Fig. 3A, B, C and **Table S3**), respectively. A total of 14 differential metabolites were shared in both groups (Fig. 3C). The 14 shared differential metabolites were enriched in pathways including febrile seizures (*P* = *P* = 1.96E-2), GABA-transaminase deficiency (*P* = 1.96E-2), and spinocerebellar degeneration (*P* = 2.93E-2) (Fig. 3D). Notably, GABA levels were decreased in both *PCCB*-G1 (decreased by 59%, *P* = 3.42E-03) and *PCCB*-G2 hFOs (decreased by 25%, *P* = 8.62E-03) when compared with control hFOs.

### PCCB knockdown in hFOs decreases GABA level by reducing tricarboxylic acid cycle and leads to mitochondrial dysfunction

**Figure 4:**
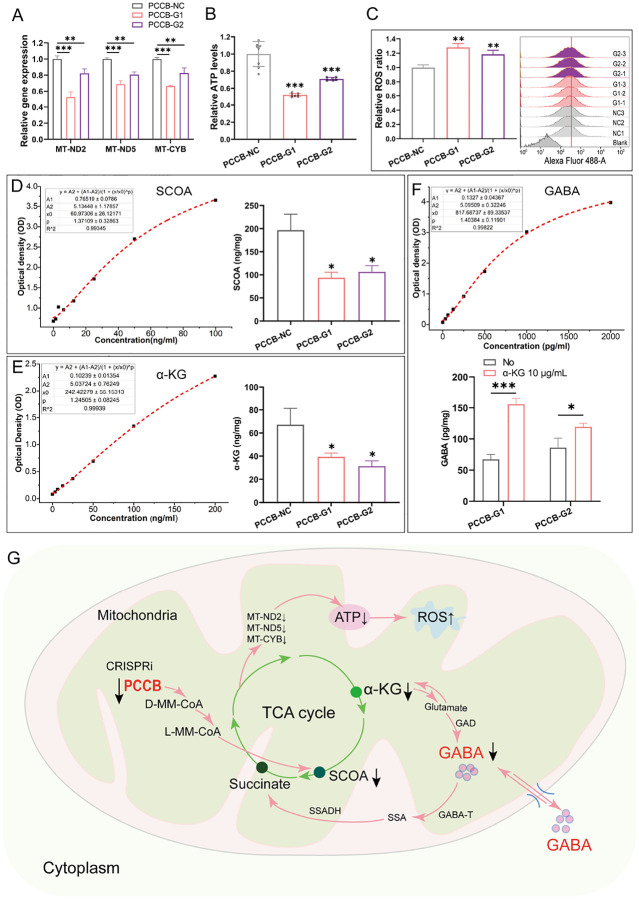
PCCB knockdown leads to mitochondrial dysfunction in hFOs. A RT-qPCR analysis revealed a reduction in the expression of mitochondria genes in PCCB knockdown hFOs. B, C Decreased ATP synthesis (B) and increased ROS content (C) were observed in PCCB knockdown hFOs via ATP assay and ROS assay. D, E ELISA assay validated a reduction in the content of Succinyl-CoA (SOCA) (D) and α-ketoglutarate (α-KG) (E) in PCCB knockdown hFOs. F Adding α-KG (10 μg/ml) into the culture medium of PCCB knockdown hFOs restored the GABA level. G Schematic diagram for the pathway that PCCB knockdown decreased GABA levels. GAD, glutamate decarboxylase; GABA-T, GABA transaminase; SSA, Succinic semialdehyde; SSADH, succinic semialdehyde dehydrogenase; D-MM-CoA, D-MethylMalonyl-CoA; L-MM-CoA, L-MethylMalonyl-CoA;↓ downregulated of expression;↑ T, upregulated of expression. The two-tailed student’s t-test was used to assess difference between the *PCCB-NC* and *PCCB-G1* or *PCCB-G2* group. *P< 0.05, **P< 0.01, ***P< 0.001.

*PCCB* encodes the β subunit of the propionyl-CoA carboxylase, a mitochondrial enzyme involved in the catabolism of propionyl-CoA^28^. *PCCB* mutation has been reported to impair mitochondrial energy metabolism by disrupting the tricarboxylic acid (TCA) cycle^16^. We would expect mitochondrial dysfunction caused by *PCCB* knockdown. Indeed, we found several mitochondrial genes that function in cellular oxidative phosphorylation, including *MT-ND2, MT-ND5*, and *MT-CYB*, were downregulated in both *PCCB*-G1 and *PCCB*-G2 hFOs (**Table S2**), which was further validated by the RT-qPCR analysis (Fig. 4A). Adenosine triphosphate (ATP) and reactive oxygen species (ROS) detection assays showed that *PCCB* knockdown reduced ATP generation and increased ROS levels in hFOs (Fig. 4B, C), indicating the mitochondrial dysfunction caused by *PCCB* knockdown.

Since GABA metabolism involves a route from a-ketoglutarate (α-KG) generated by the TCA cycle to succinate via glutamate, GABA, and succinic semialdehyde^29,30^, we examined whether *PCCB* knockdown decreased GABA level by inhibiting the TCA cycle. We performed enzyme linked immunosorbent assay (ELISA) and confirmed that *PCCB* knockdown decreased succinyl-CoA (SCOA) and a-KG (Fig. 4D, E), two key metabolites that connect the GABA shunt and TCA cycle^29,30^. Considering that α-KG is an upstream metabolite that could be converted to GABA, we added a-KG (10 μg/ml) into culture media of hFOs and found a restored GABA level (Fig. 4F) in *PCCB* knockdown hFOs. These results indicated that *PCCB* knockdown decreased GABA level by reducing TCA cycle (Fig. 4G).

### PCCB knockdown in hFOs leads to abnormal dectrophysiological activities

**Figure 5:**
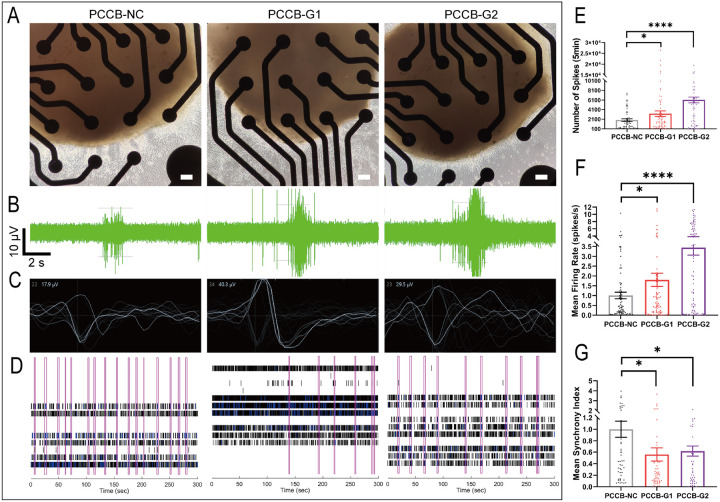
MEA assay after *PCCB* knockdown in hFOs. A Bright fields of hFOs cultured in 24-well MEA plate. Scale bar, 100 pm. B Representative burst traces for individual electrodes recorded from hFOs. C Schematic diagram of a single unit event. D Raster plot diagram of synchronized burst activity. Each pink box represents a synchronized burst. E, F, and G *PCCB* knockdown increased the number of spikes (E) and mean neuron firing rate (F), but reduced synchronized burst activity in hFOs (G). Data were shown with Mean ± SEM (averaged organoids N = 8). The two-tailed student’s t-test was used to assess difference between the *PCCB*-NC and *PCCB*-G1 or *PCCB*-G2 group. *P< 0.05, ****P< 0.0001.

Since GABA, the major inhibitory neurotransmitter in the brain^31^, was decreased in *PCCB* knockdown hFOs, we used multielectrode array (MEA) recording assay to test whether *PCCB* knockdown affected neuroactivities. The *PCCB* knockdown and control hFOs at day 160 were seeded on Matrigel-coated 24-well MEA plate (Fig. 5A). After 7 days of culture, electroactivities of hFOs were recorded (Fig. 5B, C, D). We found that *PCCB* knockdown in hFOs led to increased number of spikes (Fig. 5E) and mean neuron firing rate (Fig. 5F), suggesting a hyper neuroactivity after *PCCB* knockdown in hFOs. However, *PCCB* knockdown decreased the synchronization of the neural network (Fig. 5G). Since hyper neuroactivity and decreased synchronization of neural network in SCZ brains have been reported by the electroencephalography and magnetoencephalography^32^, these results supported that *PCCB* knockdown led to abnormal electrophysiological activities that link to SCZ phenotypes.

## Discussion

SCZ is a polygenic psychiatric disorder with risk contributed by multiple genes. Identifying genes whose expression is associated with SCZ risk by TWAS is a powerful approach to prioritize SCZ risk genes. By integrating multiple published datasets from TWAS, gene coexpression, and differential gene expression analysis, we prioritized *PCCB* as a reliable SCZ risk gene. *PCCB* is a gene encoding p subunit of propionyl-coA carboxylase enzyme^28^, defect of which has been reported as a cause of propionic acidemia^33^. Though several case reports have shown neuropathological symptoms including autistic features^34^ in propionic acidemia^35,36^, how *PCCB* deficiency led to neuropathology is largely unknown. Moreover, what roles *PCCB* plays in the etiology of SCZ has not been investigated.

To investigate *PCCB’s* contribution to SCZ risk, we performed RNA-seq analysis and identified that *PCCB* knockdown in U2F hFOs affected expression of genes related to multiple neuronal functions and GABAergic synapse pathway. To confirm the RNA-seq results, we generated hFOs using another hiPSC line (ACS-1011) (**Fig. S4A, B**), finding that *PCCB* knockdown also led to decreased expression of GABA receptor genes, including *GABRA1, GABRA2, GABRB2*, and *GABRB3* (**Fig. S4C**), as detected in U2F hFOs.

The downregulated GABAergic synapse pathway caused by *PCCB* knockdown attracted our attention, since GABAergic system dysfunction plays important roles in SCZ etiology^37,38^. We performed the metabolomic analysis and confirmed the decreased GABA levels in *PCCB* knockdown hFOs. The following electrophysiological analysis showed that *PCCB* knockdown led to hyper neuroactivity and decreased synchronization of neural network activities, cellular phenotypes reported to be associated with SCZ risk^32,39^. Through the hFOs-based multi-omics analyses, we revealed the impacts of *PCCB* in neural functions and its connection to SCZ etiology, highlighting that *PCCB* may contribute to SCZ etiology through regulating the GABAergic system.

Since the GABA shunt connected the GABA metabolism pathway and TCA cycle^29,30^, we expected that *PCCB* regulates GABAergic system by affecting TCA cycle. As expected, *PCCB* knockdown lead to reduced production of SCOA and α-KG in TCA cycle. The α-KG produced from TCA cycle could be converted into SCOA or serve as a source for GABA synthesis^40^, the decrease of α-KG may be responsible for the reduced production of GABA. On the other hand, *PCCB* knockdown lead to the reduction of SCOA, which may exacerbate the entry of GABA into the TCA cycle through GABA shunt pathway and further reduced GABA content in the cytoplasm^40^. Overall, *PCCB* knockdown decreased GABA levels by reducing the content of α-KG and SCOA in TCA cycle (Fig. 4G). As mitochondrial dysfunction has been reported to be associated with GABA dysfunction^41^ and etiology of SCZ^42,43^, our study provided evidence how mitochondrial dysfunction may contribute to SCZ risk.

In addition to mitochondrial dysfunction, one of the major effects of *PCCB* defect is the cellular accumulation of propanoic acid, propionyl carnitine, and other metabolites. Indeed, we did observe a dramatic increase of propanoic acid or propionyl carnitine in *PCCB* knockdown hFOs (**Table S3**). Interestingly, hFOs exposed to propanoic acid (3.5 uM) led to significantly decreased expression of GABA receptor genes *(GABRA1, GABRA2, GABBR2*, and *GABBR3)* (**Fig. S5**), which is consistent with those observed in *PCCB* knockdown hFOs. These results suggested that the accumulation of propanoic acid may mediate the effects of *PCCB* knockdown, and partially explain how propionic acidemia could lead to neuropathological symptoms. These results also suggested the potential effects of short-chain fatty acids on SCZ risk, since short-chain fatty acids including propanoic acid, acetic acid, and butyric acid were found to be upregulated in serum of SCZ patients^44^.

This study reveals the connection between *PCCB* and SCZ risk, some limitations also existed. First, *PCCB* knockdown affects multiple types of synapses, including GABAergic, glutamatergic, dopaminergic, and cholinergic synape, as revealed by RNA-seq analysis. The cell-type specific effects of *PCCB* knockdown is unclear. Further investigations such as RNA-seq and other omics analysis at single-cell level are needed. Second, we showed that SCZ-associated SNP rs35874192, an eQTL SNP for *PCCB*, affected transcriptional activities. But whether SNP rs35874192 could affect *PCCB* expression in-vivo remains unclear. In the future, using CRISPR-Cas9 gene editing to confirm the regulatory effects of SNP rs35874192 on *PCCB* expression is needed.

In summary, this study used hFOs-based multi-omics analyses and revealed connection between *PCCB* and SCZ, highlighting that *PCCB* may contribute to SCZ etiology through regulating the GABAergic system and mitochondrial function.

## Methods

### Prioritization of SCZ risk genes and SNPs

We combined the published results from TWAS^8–11^, MR-JTI^15^, and SMR^2,10,12,13^ analyses to prioritize SCZ risk genes with sufficient supporting evidence. We also checked whether the prioritized genes are located in SCZ risk-associated gene coexpression modules or differentially expressed in postmortem brains of SCZ patients.

To prioritize SCZ risk SNPs, we first collected top SNPs in the TWAS analysis. We then retrieved SNPs in LD (r^2^ ≥ 0.6, European population genome) with the top SNPs. We prioritized candidate causal SNPs that likely affect gene expression in the brain using the following criteria: 1) candidate SNPs are eSNPs for the SCZ risk genes in the brain based on the BrainSeq^24^, GTEx^23^, or PsychENCODE eQTL data^45^; 2) SNPs are located within chromatin open regions based on ATAC-seq data from the PsychENCODE consortium; 3) SNPs are located in genomic regions predicted as enhancers or promoters in human brain tissues or neural cell cultures based on the Roadmap Epigenomics data^22^.

### Cell Culture

Two hiPSC lines (U2F and ACS-1011) used in this study were derived from healthy individuals. The U2F hiPSCs was obtained from the Cellapy Technology (Beijing, China), and the ACS-1011 was obtained from the American type culture collection (ATCC). Pluripotency and karyotype of U2F hiPSCs were confirmed by immunofluorescence and karyotype analysis as shown in our previous studies^46,47^. The hiPSCs were cultured in Matrigel (Corning, 354277)-coated plate and supplemented with the mTeSR Plus medium (STEMCELL Technologies, 05825) and 1% penicillin/streptomycin (Gibco, 10378016).

As described in our previous study^46^, hNPCs were induced from U2F hiPSCs using the STEMdiff^™^ Neural Induction Medium (STEMCELL Technologies, 05835). The hNPCs were cultured in Matrigel-coated plate and maintained in the STEMdiff^™^ Neural Progenitor Medium (STEMCELL Technologies, 05833).

The SH-SY5Y neuroblastoma cells were cultured in high-glucose DMEM (Gibco, C11995500BT) supplemented with 10% fetal bovine serum (Gibco, A3161001C) and 1% penicillin/streptomycin.

### DLRA

About 50 bp DNA sequence (**Table S4**) flanking the *PCCB* eSNP was synthesized and cloned into the pGL3-basic vector using the restriction enzymes Kpnl and Xhol (New England BioLabs). The *PCCB* promoter sequence (~ 600 bp) was amplified from the genomic DNA using the PCR primers (F, 5’-CCGCTCGAGTTTGAATCCTGGCCAACCAC-3’; R, 5’-CCC AAGCTTTGCTAAAGCGTGGGTACGG-3’) and Phanta^®^Max Super-fidelity DNA Polymerase (Vazyme, P505-d1). The amplified *PCCB* promoter was then cloned into downstream of the eSNP-containing DNA fragment using the restriction enzymes Xhol and Hind III (New England BioLabs). DLRA was performed in both hNPCs and SH-SY5Y cells. For DLRA, 1× 10^5^ cells per well were plated into 24-well plate. After 24 hours of culture, 500 ng recombinant pGL3-basic luciferase reporter vector and 20 ng PRL-TK Renilla internal control vector for each well were co-transfected into cells using Lipofectamine^™^ 3000 (Invitrogen, L3000015). 36 hours post transfection, the Firefly and Renilla luciferase activities were measured on the LumiPro luminescence detector (Lu-2021-C001) using the DLRA kit (Vazyme, DL101–01). Experiments were conducted in three biological replicates.

### Establishment of PCCB knockdown and control hiPSCs

We used CRISPRi to establish *PCCB* knockdown and control hiPSCs. Two gRNA sequences targeting *PCCB* (*PCCB*-G1, 5’-GCATTACGGGTGGCGGCGGT-3’; *PCCB*-G2, 5’-GCGTACTCAGGTGCGCCGGT-3’) were designed using the online tool CRISPR-ERA (http://crispr-era.stanford.edu/). The PCCB-targeting gRNA or control gRNA sequence (5’-GCGCCAAACGTGCCCTGACGG-3’)^48^ were synthesized and cloned into the lentiviral vector pLV-hU6-sgRNA-hUbC-dCas9-KRAB-T2a-Puro. The constructed vectors were then used for lentiviral package (ObiO Technoliges, China). For viral infection in hiPSCs, when cell confluence reached 40–50% in 12-well plate, cells were cultured in 0.6 mL medium and incubated with the lentiviruses for 24 h. The culture medium was fully replaced in the next day. 48 hours post viral infection, cells were treated with 0.5–1 μg/ml puromycin for 3–7 days to kill cells that were not infected by the lentiviruses. *PCCB* knockdown efficiency was confirmed by RT-qPCR analysis.

### Rt-qpcr Analysis

Total RNA was used to generate complementary DNA using HiScript III RT SuperMix for qPCR (+ gDNA wiper) (Vazyme, R323–01). RT-qPCR assay was performed using ChamQ SYBR qPCR Master Mix (Vazyme, Q711–02) on the Real-Time PCR System (Roche, LightCycler 480 II). GAPDH was used as the internal reference gene. At least three technical replicates were used in the RT-qPCR analysis. RT-qPCR primers are provided in **Table S4**.

### Generation Of Hfos

The established *PCCB* knockdown and control hiPSCs were used to generate hFOs using the STEMdiff Dorsal Forebrain Organoid Differentiation Kit (STEMCELL Technologies, 08620) based on the manufacturer’s instructions with some modifications. Briefly, hiPSCs were dissociated into single cells using Accutase solution (Sigma-Aldrich, A6964) on day 0. 1× 10^4^ cells per well were then plated into 96-well round-bottom ultra-low attachment plate (Corning, 7007) and fed with 50 μL Forebrain Organoid Formation Medium supplemented with 1× Penicillin/Streptomycin and 10 μM Y27632 (Selleck, SCM075). On day 3, each well was gently added with 50 μL fresh Forebrain Organoid Formation Medium without Y27632. On day 6, the medium was replaced with the Forebrain Organoid Expansion Medium. On day 25, the Forebrain Organoid Expansion Medium was replaced with the Forebrain Organoid Differentiation Medium. From day 43, organoids were cultured in the Forebrain Organoid Maintenance Medium. hFOs were characterized using immunostaining as described in our previous studies^46,47^.

### Bulk Organoid Rna-seq And Data Analysis

Two hFOs from *PCCB* knockdown or control group were randomly selected and pooled together as one mixed sample (day 60, N = 5 in each group) for total RNA extraction using the miRNeasy Mini Kit (Qiagen, 217004). RNA quality was evaluated on the Agilent 2100 Bioanalyzer system. RNA samples with RNA integrity numbers over 7 were used for RNA-seq (150 bp, paired-end) on the Illumina NovaSeq 6000 system.

Raw RNA-seq data were filtered to get clean reads using FastQC (v0.20.0). The clean reads were aligned to the human genome hg38 using STAR (v2.7.9a). Gene expression quantification was conducted using RSEM (v1.3.0) based on Gencode v40 comprehensive gene annotation. The filterByExpr function in the edgeR package (v3.36.0) was used to filter out low-expression genes. The sva function in the SVA package (v3.42.0) was used to estimate batch effect and other artifacts. Differential gene expression analysis between the *PCCB* knockdown and control group was performed using the DEseq2 package (v1.34.0). *P* values were adjusted using the Benjamini-Hochberg method. To annotate the functions of DEGs, we used the online tool WebGestalt2019 (http://www.webgestalt.org/) to perform GO and KEGG pathway enrichment analysis.

### Ppi Analysis

The STRING database (v11.5) (http://www.string-db.org/) was used to construct a high-confidence (interaction score > 0.7) PPI network for the PCCB-induced DEGs. Active interaction sources included text-mining, experiments, databases, coexpression, neighborhood, gene fusion, and co-occurrence. The PPI network was visualized using Cytoscape (v 3.9.1). CytoHubba^49^, a Cytoscape plugin, was used to explore hub nodes in the PPI network.

### Metabolomic Analysis Of Bulk Hfos

For metabolomic analysis, three hFOs (day 60) in *PCCB* knockdown or control group were randomly selected and pooled together as one mixed sample. Five mixed samples in each group were then used for HM400 metabolomic analysis (Beijing Genomics Institute, China). Briefly, hFOs or quality control samples were lysed in 140 μL 50% water/methanol solution. The lysate was centrifuged (18000g, 4°C, 20 min) to get the supernatant. The supernatant was used for derivatization reaction and then centrifuged at 4000g, 4°C, 10 min. The supernatant was further used for high performance liquid chromatography tandem mass spectrometer (LC-MS/MS) analysis on the SCIEX QTRAP 6500 + LC-MS/MS system. Parameters of liquid chromatographic column were BEH C18 (2.1 mm × 10 cm, 1.7um, Waters). The parameter of mass spectrometry was ESI+/ESI−. Content of metabolites (μmol/g) was quantified using the HMQuant software based on the formula (C*0.14/m), where C represents the calculated concentration (μmol/L) and m represents the sample weight (mg). Two-tailed t-test was used to identify differential metabolites between *PCCB* knockdown and control hFOs. Functional annotation for the differentially expressed metabolites was performed using the online tool MetaboAnalyst (https://www.metaboanalyst.ca/).

### Atp Assay

ATP assay was used to examine the effects of *PCCB* knockdown on ATP production using the ATP assay kit (Beyotime, S0026). At day 60, *PCCB* knockdown or control hFOs were washed twice with PBS and lysed with 200 μL lysis buffer per well. The lysate was centrifuged at 12,000g for 5 min at 4°C. The supernatant was used to detect ATP levels on the LumiPro luminescence detector (Lu-2021-C001). Experiments were conducted in more than three biological replicates.

### Ros Assay

The ROS Assay Kit (Beyotime, S0033S) was used to measure ROS levels in *PCCB* knockdown and control hFOs. Briefly, hFOs (day 60, three hFOs were randomly pooled together as one mixed sample) were dissociated into single cells using Accutase. After centrifuging at 1500 rpm for 5 minutes, cells were resuspended with pre-warmed serum-free DMEM-F12. The collected cells were incubated with 10 μM DCFH-DA fluorescent probes in serum-free DMEM-F12 for 20–30 min at 37°C, 5% CO_2_. 1× 10^4^ cells were analyzed on the flow cytometry (BD FACSAria II). Experiments were conducted in four biological replicates.

### Elisa Assay

The ELISA Assay Kits (CAMILO, Nanjing, China) were used to measure SOCA (Cat No. 2H-KMLJh315292), a-KG (Cat No. 2H-KMLJh313735) and GABA (Cat No. 2H-KMLJh310295) levels in *PCCB* knockdown and control hFOs according to the manufacturer’s instructions. Briefly, protein was extracted from hFOs (day 60, two hFOs were randomly pooled together as one mixed sample) using RIPA buffer (Beyotime, P0013B) containing 1 % protease inhibitor. The lysate was centrifuged at 12,000g for 10 min at 4°C. Protein concentration was determined using the BCA protein assay Kit (Vazyme, E112–01). The supernatant was used to detect SCOA, α-KG and GABA. We also used ELISA to determine the restoration of GABA levels in *PCCB* knockdown hFOs following exogenous α-KG treatment (incubating the hFOs with 10 μg/ml α-KG for 3 hours at 37°C, 5% CO_2_). Experiments were conducted in four biological replicates.

### Mea Assay

We used an MEA assay to evaluate the effects of *PCCB* knockdown on electrophysiological properties in hFOs. At day 160, hFOs were cultured in the Matrigel-coated 24-well MEA plate with one organoid seeded in each well. After 7 days of culture, electro activities were recorded using the Axion Biosystems. Each recording duration was 5 min. The MEA data including the number of spikes, burst frequency, and network synchronization were analyzed using the software Axis Navigator 3.6.2.

### Statistical analysis

Data were analyzed with GraphPad Prism 9.0.0 and presented as Mean ± SEM. A two-tailed t-test was used to assess the difference between two groups. The hypergeometric test was used to assess the enrichment between *PCCB*-induced DEGs and SCZ-related gene sets. *P* values in the differential gene expression analysis were adjusted for multiple testing using the Benjamini-Hochberg method. For all statistical analyses, a *P* value less than 0.05 is considered statistically significant.

## Figures and Tables

**Table 1:** Prioritized top three SCZ risk genes. ↓, Downregulation in SCZ; ↑, Upregulation in SCZ;

Genes	MR-JTI	TWAS				SMR			Differential expression	Coexpression module
		FUSION	PrediXcan	FUSION	FUSION					
	Wu, Y., et al 2021	Hall, L. S. et al., 2020	Huckins, L. M. et al., 2019	Gusev, A. et al., 2018	Gandal, M. J. et al., 2018	Yang, Z. et al., 2020	Gandal, M. J. et al., 2018	Li, Z. et al., 2017	Goncalves, V. F. et al., 2018	Gandal, M. J. et al., 2018
PCCB	*P*_Bonferroni_ = 3.17E-11	*P*_TWAS_ = 5.39E-12	*P* = 2.05E-08	*P*_TWAS_ = 3.00E-10	*P*_Bonferroni_ = 2.42E-05	-	*P*_SMR_ = 3.74E-10	*P*_SMR_ = 4.17E-15	↓ *P*=0.01	↓ FDR = 6.71E-03
GATAD2A	*P*_Bonferroni_ = 1.87E-08	*P*_TWAS_ = 8.67E-11	*P* = 2.18E-10	*P*_TWAS_ = 9.00E-07	*P*_Bonferroni_ = 6.98E-09	*P*_SMR-multi_ < 1.00E-05	*P*_SMR_ = 2.21E-10	-	-	↑ FDR = 5.03E-03
GNL3	*P*_Bonferroni_ = 1.58E-18	-	*P* = 1.39E-11	*P*_TWAS_ = 6.00E-07	*P*_Bonferroni_ = 8.24E-03	*P*_SMR-multi_ < 1.00E-05	*P*_SMR_ = 4.71E-09	*P*_SMR_ = 4.53E-13	-	-

**Table 2:** Prioritized eQTL SNPs for PCCB.

Source	References	Best GWAS SNP	P value	LD eSNP	LDCr^2^)	Ref/Alt	Chromatin states (Core 15-state model)	PsychEncode ATAC-seq Peak
MR-JTI	Wu, Y et al., Mol Neurobiol (2021)	-	*P*_Bonferroni_ = 3.17E-11	-	-	-	-	-
TWAS	Hall, L. S. et al., Hum Mol Genet (2020)	rs7432375	*P*_twas_ = 5.39E-12	rs6791142	0.81	T/C	7_Enh	YES
				rs35874192	0.62	G/C	1_TssA	YES
				rs900818	0.63	C/T	1_TssA, 2_TssAFlnk	YES
	Gusev, A. et al., Nat Genet (2018)	rs7432375	*P*_twas_ = 5.27E-11	rs7616204	0.64	C/T	7_Enh	YES
	Huckins, L. M. et al., Nat Genet (2019)	-	*P*_twas_ = 2.05E-08	-	-	-	-	-
	Gandal, M. J. et al., Science (2018)	rs7427564	*P*_twas_ = 1.71E-09	rs6791142	0.73	T/C	7_Enh	YES
SMR	Gandal, M. J. et al., Science (2018)	rs527888	*P*_SMR_ = 3.74E-10	rs570621	0.95	A/G	7_Enh	YES
				rs7349597	0.69	C/T	1_TssA, 2_TssAFlnk, 7_Enh	YES
	Li, Z. et al., Nat Genet. (2017)	rs66691851	*P*_SMR_ = 4.17E-15	rs7349597	0.6	C/T	1_TssA, 2_TssAFlnk, 7_Enh	YES
				rs570621	0.81	A/G	7_Enh	YES
				rs6791142	0.6	T/C	7_Enh	YES

1_TssA, Active transcription start site (TSS); 2_TssAFlnk, Flanking active TSS; 7_Enh, Enhancers; Ref/Alt, Reference/Alternative allele.

## Data Availability

RNA-seq data are deposited in Gene Expression Omnibus (Accession number: GSE226233). Raw MEA data and metabolomic data are available on request. All other data associated with this study are shown in the manuscript or Supplementary Materials.

## References

[1] Hilker R. Heritability of Schizophrenia and Schizophrenia Spectrum Based on the Nationwide Danish Twin Register. Biol. Psychiatry 83, 492–498, doi:10.1016/j.biopsych.2017.08.017 (2018). 28987712

[2] Trubetskoy V. Mapping genomic loci implicates genes and synaptic biology in schizophrenia. Nature 604, 502–508, doi:10.1038/s41586-022-04434-5 (2022). 35396580 PMC9392466

[3] Schizophrenia Working Group of the Psychiatric Genomics, C. Biological insights from 108 schizophrenia-associated genetic loci. Nature 511, 421–427, doi:10.1038/nature13595 (2014). 25056061 PMC4112379

[4] Maurano M. T. Systematic Localization of Common Disease-Associated Variation in Regulatory DNA. Science 337, 1190 (2012). 22955828 10.1126/science.1222794 PMC3771521

[5] Degner J. F. DNasel sensitivity QTLs are a major determinant of human expression variation. Nature 482, 390–394 (2012). 22307276 10.1038/nature10808 PMC3501342

[6] Huo Y., Li S., Liu J., Li X. & Luo X. J. Functional genomics reveal gene regulatory mechanisms underlying schizophrenia risk. Nat. Commun. 10, 670, doi:10.1038/s41467-019-08666-4 (2019). 30737407 PMC6368563

[7] Boyle E. A., Li Y. I. & Pritchard J. K. An Expanded View of Complex Traits: From Polygenic to Omnigenic. Cell 169, 1177–1186, doi:10.1016/j.cell.2017.05.038 (2017). 28622505 PMC5536862

[8] Gusev A. Transcriptome-wide association study of schizophrenia and chromatin activity yields mechanistic disease insights. Nat Genet 50, 538–548, doi:10.1038/s41588-018-0092-1 (2018). 29632383 PMC5942893

[9] Hall L. S. A transcriptome-wide association study implicates specific pre- and post-synaptic abnormalities in schizophrenia. Hum Mol Genet 29, 159–167, doi:10.1093/hmg/ddz253 (2020). 31691811 PMC7416679

[10] Gandal M. J. Transcriptome-wide isoform-level dysregulation in ASD, schizophrenia, and bipolar disorder. Science 362, doi:10.1126/science.aat8127 (2018). PMC6443102 30545856

[11] Huckins L. M. Gene expression imputation across multiple brain regions provides insights into schizophrenia risk. Nat. Genet. 51, 659–674, doi:10.1038/s41588-019-0364-4 (2019). 30911161 PMC7034316

[12] Li Z. Genome-wide association analysis identifies 30 new susceptibility loci for schizophrenia. Nat Genet 49, 1576–1583, doi:10.1038/ng.3973 (2017). 28991256

[13] Yang Z. The genome-wide risk alleles for psychiatric disorders at 3p21.1 show convergent effects on mRNA expression, cognitive function, and mushroom dendritic spine. Mol. Psychiatry 25, 48–66, doi:10.1038/s41380-019-0592-0 (2020). 31723243

[14] Wu Y., Yu X. L., Xiao X., Li M. & Li Y. Joint-Tissue Integrative Analysis Identified Hundreds of Schizophrenia Risk Genes. Mol. Neurobiol. 59, 107–116, doi:10.1007/s12035-021-02572-x (2022). 34628600

[15] Zhou D. A unified framework for joint-tissue transcriptome-wide association and Mendelian randomization analysis. Nat. Genet. 52, 1239–1246, doi:10.1038/s41588-020-0706-2 (2020). 33020666 PMC7606598

[16] Chapman K. A. Propionyl-CoA carboxylase pcca-1 and pccb-1 gene deletions in Caenorhabditis elegans globally impair mitochondrial energy metabolism. J Inherit Metab Dis 41, 157–168, doi:10.1007/s10545-017-0111-x (2018). 29159707 PMC5832583

[17] Stathopoulos S. DNA Methylation Associated with Mitochondrial Dysfunction in a South African Autism Spectrum Disorder Cohort. Autism Res 13, 1079–1093, doi:10.1002/aur.2310 (2020). 32490597 PMC7496548

[18] Lancaster M. A. Cerebral organoids model human brain development and microcephaly. Nature 501, 373–379, doi:10.1038/nature12517 (2013). 23995685 PMC3817409

[19] Goncalves V. F. A Comprehensive Analysis of Nuclear-Encoded Mitochondrial Genes in Schizophrenia. Biol. Psychiatry 83, 780789, doi:10.1016/j.biopsych.2018.02.1175 (2018). PMC7168759 29628042

[20] Fromer M. Gene expression elucidates functional impact of polygenic risk for schizophrenia. Nat. Neurosci. 19, 1442–1453, doi:10.1038/nn.4399 (2016). 27668389 PMC5083142

[21] Psych E. C. The PsychENCODE project. Nat. Neurosci. 18, 1707–1712, doi:10.1038/nn.4156 (2015). 26605881 PMC4675669

[22] Kundaje A. Integrative analysis of 111 reference human epigenomes. Nature 518, 317–330 (2015). 25693563 10.1038/nature14248 PMC4530010

[23] Consortium, G. T. The GTEx Consortium atlas of genetic regulatory effects across human tissues. Science 369, 1318–1330, doi:10.1126/science.aaz1776 (2020). 32913098 PMC7737656

[24] Jaffe A. E. Developmental and genetic regulation of the human cortex transcriptome illuminate schizophrenia pathogenesis. Nat. Neurosci. 21, 1117–1125, doi:10.1038/s41593-018-0197-y (2018). 30050107 PMC6438700

[25] Liao Y., Wang J., Jaehnig E. J., Shi Z. & Zhang B. WebGestalt 2019: gene set analysis toolkit with revamped UIs and APIs. Nucleic Acids Res. 47, W199–W205, doi:10.1093/nar/gkz401 (2019). 31114916 PMC6602449

[26] Kathuria A. Transcriptomic Landscape and Functional Characterization of Induced Pluripotent Stem Cell-Derived Cerebral Organoids in Schizophrenia. JAMA Psychiatry 77, 745–754, doi:10.1001/jamapsychiatry.2020.0196 (2020). 32186681 PMC7081156

[27] Watanabe K., Taskesen E., van Bochoven A. & Posthuma D. Functional mapping and annotation of genetic associations with FUMA. Nat. Commun. 8, 1826, doi:10.1038/s41467-017-01261-5 (2017). 29184056 PMC5705698

[28] Jiang H., Rao K. S., Yee V. C. & Kraus J. P. Characterization of four variant forms of human propionyl-CoA carboxylase expressed in Escherichia coli. J. Biol. Chem. 280, 27719–27727, doi:10.1074/jbc.M413281200 (2005). 15890657

[29] Morland C. Propionate enters GABAergic neurons, inhibits GABA transaminase, causes GABA accumulation and lethargy in a model of propionic acidemia. Biochem J 475, 749–758, doi:10.1042/BCJ20170814 (2018). 29339464

[30] Kim K. & Yoon H. Gamma-Aminobutyric Acid Signaling in Damage Response, Metabolism, and Disease. International Journal of Molecular Sciences 24, doi:10.3390/ijms24054584 (2023). PMC10003236 36902014

[31] McCormick D. A. GABA as an inhibitory neurotransmitter in human cerebral cortex. J. Neurophysiol. 62, 1018–1027, doi:10.1152/jn.1989.62.5.1018 (1989). 2573696

[32] Uhlhaas P. J. & Singer W. Abnormal neural oscillations and synchrony in schizophrenia. Nat. Rev. Neurosci. 11, 100–113, doi:10.1038/nrn2774 (2010). 20087360

[33] Ugarte M. Overview of mutations in the PCCA and PCCB genes causing propionic acidemia. Hum. Mutat. 14, 275–282, doi:10.1002/(SICI)1098-1004(199910)14:4<275∷AID-HUMU1>3.0.CO;2-N (1999). 10502773

[34] Witters P. Autism in patients with propionic acidemia. Mol. Genet. Metab. 119, 317–321, doi:10.1016/j.ymgme.2016.10.009 (2016). 27825584

[35] Schreiber J. Neurologic considerations in propionic acidemia. Mol. Genet. Metab. 105, 10–15, doi:10.1016/j.ymgme.2011.10.003 (2012). 22078457

[36] Cao L. X. Neuropathological report of propionic acidemia. Neuropathology, doi:10.1111/neup.12861 (2022). 36102083

[37] Jahangir M., Zhou J. S., Lang B. & Wang X. P. GABAergic System Dysfunction and Challenges in Schizophrenia Research. Front Cell Dev Biol 9, 663854, doi:10.3389/fcell.2021.663854 (2021). 34055795 PMC8160111

[38] Marques T. R. GABA-A receptor differences in schizophrenia: a positron emission tomography study using [(11)C]Ro154513. Mol Psychiatry 26, 2616–2625, doi:10.1038/s41380-020-0711-y (2021). 32296127 PMC8440185

[39] Schmidt M. J. & Mirnics K. Neurodevelopment, GABA system dysfunction, and schizophrenia. Neuropsychopharmacology 40, 190–206, doi:10.1038/npp.2014.95 (2015). 24759129 PMC4262918

[40] Bown A. W. & Shelp B. J. Does the GABA Shunt Regulate Cytosolic GABA? Trends Plant Sci 25, 422–424, doi:10.1016/j.tplants.2020.03.001 (2020). 32304653

[41] Kanellopoulos A. K. Aralar Sequesters GABA into Hyperactive Mitochondria, Causing Social Behavior Deficits. Cell 180, 1178–1197 e1120, doi:10.1016/j.cell.2020.02.044 (2020). 32200800

[42] Oraki Kohshour M. Association between mitochondria-related genes and cognitive performance in the PsyCourse Study. Journal of Affective Disorders 325, 1–6, doi:10.1016/j.jad.2023.01.013 (2023). 36621676

[43] Li J. Mitochondrial deficits in human iPSC-derived neurons from patients with 22q11.2 deletion syndrome and schizophrenia. Transl Psychiatry 9, 302, doi:10.1038/s41398-019-0643-y (2019). 31740674 PMC6861238

[44] Zhu F. Role of short-chain fatty acids in the gut-brain axis in schizophrenia: contribution to immune activation and pathophysiology in humans and mice. bioRxiv, 2020.2004.2011.021915, doi:10.1101/2020.04.11.021915 (2020).

[45] Wang D. Comprehensive functional genomic resource and integrative model for the human brain. Science 362, doi:10.1126/science.aat8464 (2018). PMC6413328 30545857

[46] Meng Q. Integrative analyses prioritize GNL3 as a risk gene for bipolar disorder. Mol. Psychiatry 25, 2672–2684, doi:10.1038/s41380-020-00866-5 (2020). 32826963

[47] Meng Q. Human forebrain organoids reveal connections between valproic acid exposure and autism risk. Transl Psychiatry 12, 130, doi:10.1038/s41398-022-01898-x (2022). 35351869 PMC8964691

[48] Gilbert L. A. Genome-Scale CRISPR-Mediated Control of Gene Repression and Activation. Cell 159, 647–661, doi:10.1016/j.cell.2014.09.029 (2014). 25307932 PMC4253859

[49] Chin C. H. cytoHubba: identifying hub objects and sub-networks from complex interactome. BMC Syst. Biol. 8 Suppl 4, S11, doi:10.1186/1752-0509-8-S4-S11 (2014). 25521941 PMC4290687

